# Review: Structure and mechanism of the dynein motor ATPase

**DOI:** 10.1002/bip.22856

**Published:** 2016-05-20

**Authors:** Helgo Schmidt, Andrew P. Carter

**Affiliations:** ^1^ Division of Structural StudiesMedical Research Council Laboratory of Molecular BiologyFrancis Crick AvenueCambridgeCB2 0QHUK

**Keywords:** dynein, ATPase, microtubule, mechanism

## Abstract

Dyneins are multiprotein complexes that move cargo along microtubules in the minus end direction. The largest individual component of the dynein complex is the heavy chain. Its C‐terminal 3500 amino‐acid residues form the motor domain, which hydrolyses ATP in its ring of AAA+ (ATPases associated with diverse cellular activities) domains to generate the force for movement. The production of force is synchronized with cycles of microtubule binding and release, another important prerequisite for efficient motility along the microtubule. Although the large scale conformational changes that lead to force production and microtubule affinity regulation are well established, it has been largely enigmatic how ATP‐hydrolysis in the AAA+ ring causes these rearrangements. The past five years have seen a surge of high resolution information on the dynein motor domain that finally allowed unprecedented insights into this important open question. This review, part of the “ATP and GTP hydrolysis in Biology” special issue, will summarize our current understanding of the dynein motor mechanism with a special emphasis on the recently obtained crystal and EM structures. © 2016 Wiley Periodicals, Inc. Biopolymers 105: 557–567, 2016.

## INTRODUCTION

Dyneins are a large family of motor proteins that harness the energy of ATP‐hydrolysis to move along microtubules in the minus end direction. There is one cytoplasmic dynein‐1, one cytoplasmic‐dynein‐2 and a large family of axonemal dynein isoforms.^1^ Among these different isoforms, dynein‐1 is responsible for most of the microtubule minus end directed movement in cells. It transports proteins and mRNA complexes,^2^ viruses like herpes and rabies,^3^ endosomes,^4^ mitochondria,^5^ and nuclei.^6^, ^7^ This isoform also plays important roles during mitosis, where it is involved in focusing the mitotic spindle poles,^8^ alignment of the mitotic spindle,^9^ and silencing of the kinetochore assembly checkpoint.^10^, ^11^ Dynein‐2 is involved in the assembly and maintenance of cilia.^12^ The axonemal dynein isoforms drive the beating of motile cilia and flagella.^13^

All dyneins exist as protein complexes with molecular weights of 0.7–1.8 MDa, consisting of one to three heavy chains and multiple accessory chains. The N‐terminal tail part of the heavy chain acts as an assembly platform for the accessory chains and binds cargos. In dynein‐1, but not dynein‐2,^14^ cargo binding also requires the 1.2 MDa dynactin complex.^15^ In all dynein heavy chains, the C‐terminal ∼3500 residues form the motor, an engine which consumes ATP and generates movement along microtubules.

**Figure 1:**
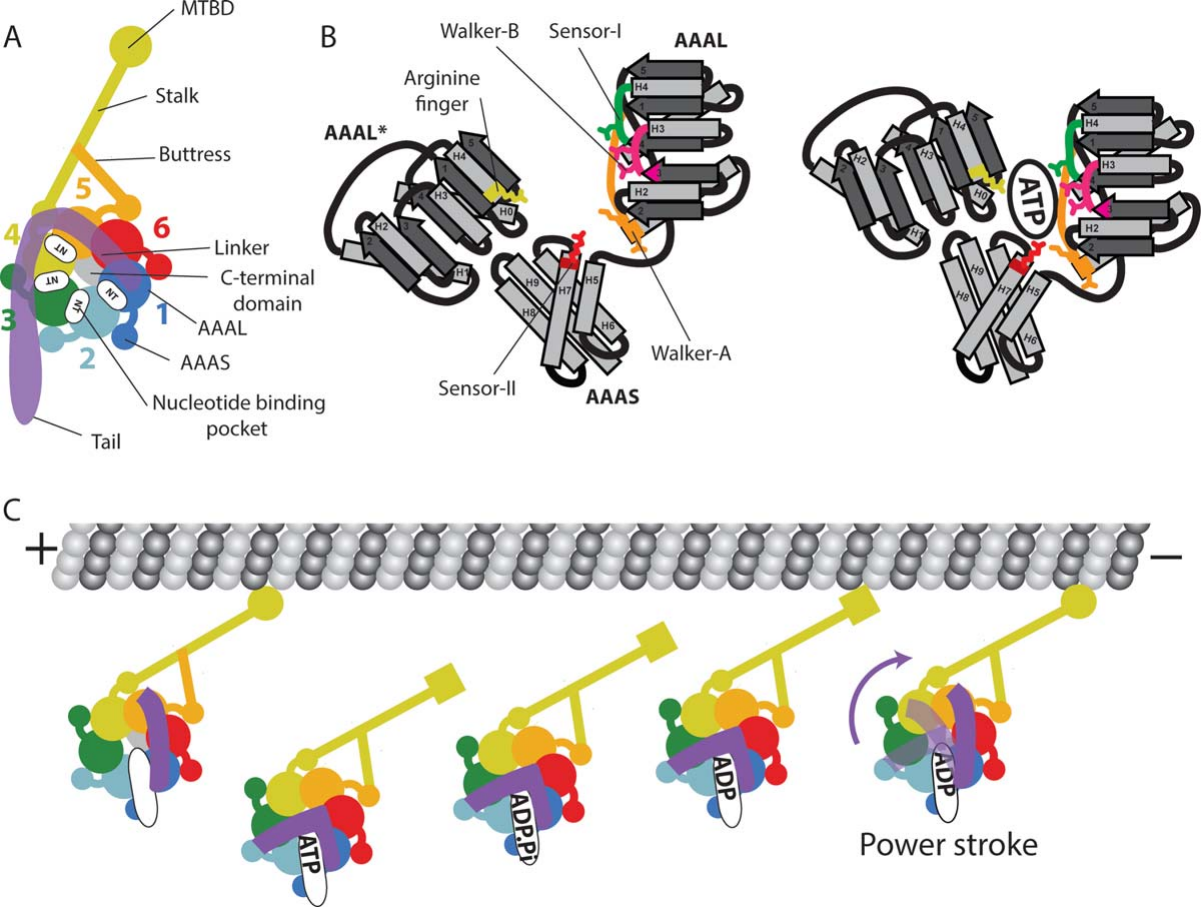
Dynein motor architecture and mechanism. A: Structural elements of the dynein motor. The ring of AAA+ domains consists of large and small subdomains (AAAL and AAAS, respectively). Each AAAL is tightly associated with AAAS of its counter clockwise neighbour (e.g. AAA2L/AAA1S). The AAA+ ring features extensions: the linker, the stalk, the buttress and the C‐terminal domain. The microtubule binding domain sits at the top of the stalk. The nucleotide binding pockets are at the interfaces between AAA+ domains. The AAA1 pocket is the main ATP‐hydrolysis site. (B) Left panel: Cartoon representation of the catalytic residues at the nucleotide binding sites. AAAL harbours the Walker‐A (orange) and Walker‐B motifs (pink) as well as the sensor‐I (green). The sensor‐II (red) and the arginine finger (yellow) are provided by AAAS and the tightly associated neighbouring AAAL (marked by an asterisk, AAAL*), respectively. Right panel: ATP binding leads to the closure of the binding site and brings the catalytic residues into close contact. Seconday structure elements are labelled. (C) Dynein motor mechanism. When the AAA1 site is empty, the dynein motor is attached to the microtubule (round MTBD) and the linker is straight. When ATP binds to AAA1, the MTBD releases from the microtubule (square MTBD) and the linker bends. After ATP‐hydrolysis, phosphate is released. Rebinding to the microtubule leads to the “power stroke”, the straightening of the linker, which is the cargo displacing step of the dynein motor cycle. The release of ADP from the AAA1 site resets the cycle.

The dynein motor contains a ring of six AAA+ domains from which four extensions emerge, the linker, the stalk, the buttress, and the C‐terminal domain^16^, ^17^, ^18^, ^19^, ^20^, ^21^ (Figure 1A). The stalk harbours the microtubule binding domain (MTBD) which interacts with the microtubule track. The AAA+ domains are split in large (AAAL) and a small (AAAS) subdomains. Each of the six dynein AAALs consists of a core of five β‐sheets and five α‐helices, which can be extended by additional inserts, mostly β‐hairpins or α‐helices.^22^ The dynein AAAS subdomains consist of a core of five α‐helices and can also contain additional polypeptide loops and helices.

In general, ATP‐hydrolysis in AAA+ proteins occurs at a tripartite interface between a AAAL, AAAS and a neighbouring AAAL using conserved catalytic residues^23^ (Figure 1B). In the first AAAL the Walker‐A motif (or P‐loop) is responsible for ATP‐binding, the Walker‐B motif activates a water molecule for ATP‐hydrolysis and the sensor‐I asparagine helps position a water molecule for the nucleophilic attack onto the ATP γ‐phosphate. The AAAS contributes a sensor‐II arginine residue which is involved in ATP‐binding. The neighbouring AAAL carries the arginine finger, which is important for stabilizing the transition state of ATP‐hydrolysis.

Out of the six AAA+ domains of the dynein motor, only the first four are able to bind nucleotide.^24^ Of these only AAA1 is strictly required for motility^25^ and harbours the complete set of catalytic residues in all dynein isoforms. The other nucleotide binding sites show a varying degree of conservation and seem to have accessory or regulatory roles for dynein motility.^22^, ^26^, ^27^ AAA5 and AAA6 have lost all catalytic residues and mainly function as a structural base for the stalk or buttress, respectively.

The ATP‐hydrolysis steps at AAA1 are coupled to the dynein motility cycle, which is characterized by conformational changes in the linker and MTBD^26,^ 27 (Figure 1C). In the absence of ATP, the linker adopts a straight conformation with its N‐terminus contacting AAA5L on the AAA+ ring. The MTBD is strongly attached to the microtubule. When ATP binds to AAA1, the MTBD loses the connection with the microtubule and the linker bends so that its N‐terminus contacts AAA2/AAA3.^25^ After ATP‐hydrolysis, the dynein motor rebinds to the microtubule, which causes the linker to undergo the powerstroke. This linker transition from a bent to a straight conformation produces the force for cargo movement.^28^, ^29^ After the powerstroke, the linker N‐terminus lies close to AAA4. Return of the linker to its original position at AAA5L correlates with the release of ADP and resets the ATP‐hydrolysis cycle. This basic mechanism can be modified by accessory ATP‐binding sites, especially AAA3,^30^ and regulators, such as Lis1,^31^ to fine tune dynein motor activity for different biological environments.

In the following sections, we discuss in detail how the ATP‐hydrolysis steps at AAA1 trigger the conformational rearrangements of the linker and MTBD, the most fundamental aspects of the dynein motor cycle. We start by analysing the structural events that occur upon ATP‐binding, describe the dynein ATP‐hydrolysis mechanism and finally discuss the events that lead to phosphate and ADP release to reset the dynein motor cycle.

## CONFORMATIONAL CHANGES IN THE DYNEIN MOTOR DOMAIN UPON ATP‐BINDING

**Figure 2:**
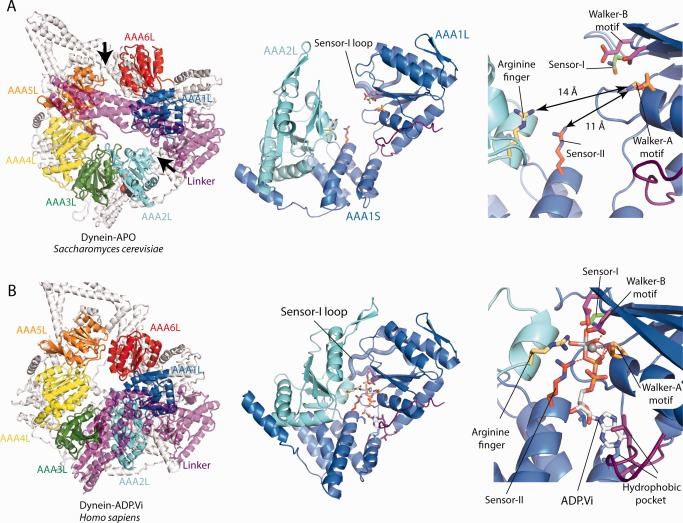
The conformation of the AAA+ ring in the dynein‐APO and dynein‐ADP.Vi state. A: Dynein‐APO. Left panel: The linker is straight and there are two gaps in the AAA+ ring, between AAA1L/AAA2L and AAA5L/AAA6L (black arrows). Middle panel: The gap between AAA1L/AAA2L opens up the AAA1 nucleotide binding site. The sensor‐I loop (thick cartoon representation) points away from AAA2L. Right panel: Enlarged view of the middle panel. The distances between the Walker‐A motif and the arginine finger and sensor‐II residues are 14 Å and 11 Å, respectively. This indicates that the AAA1 site is not competent for ATP‐hydrolysis. B: Dynein‐ADP.Vi. Left panel: The linker is bent and the gaps in the AAA+ ring have disappeared. Middle panel: The AAA1 nucleotide binding site is closed. The sensor‐I loop (thick cartoon representation) contacts AAA2L. Left panel: Enlarged view of middle panel. All catalytic residues are in close contact and in a conformation that supports ATP‐hydrolysis. Hydrophobic residues contact the adenine base of the ADP.Vi. For clarity all AAASs are shown in white colour. Colour coding of catalytic residues is the same as in figure1.

The start of the dynein motor cycle was visualized by a crystal structure^21^ and EM structures of dyneins in the absence of nucleotide (dynein‐APO).^32^, ^33^ The AAA+ ring is split into two blocks with gaps between AAA1/AAA2 and AAA5/AAA6 (Figure 2A). As a consequence, the tripartite interface between AAA1L, AAA1S and AAA2L necessary for the hydrolysis of ATP is not formed. The AAA2L arginine finger is 14 Å away from the AAA1L Walker‐A motif and the loop harbouring the sensor‐I asparagine points away from AAA2L. This indicates that this conformation of the AAA1 site is not competent for ATP‐hydrolysis. Furthermore, the position of AAA1S places the sensor‐II arginine 11 Å away from the AAA1L Walker‐A motif and opens up the adenine binding pocket between AAA1L and AAA1S, which prevents any nucleotide binding.^21^

The overall conformational changes upon AAA1 ATP‐binding can be inferred from electron microscopy studies on a dynein motor mutant deficient in ATP‐hydrolysis due to a Walker‐B E–> Q mutation.^34^ The gaps between AAA5/AAA6 and AAA1/AAA2 disappear, which leads to a more closed AAA+ ring, and the linker adopts the bent conformation. This ATP induced bending of the linker is consistent with earlier FRET studies that used a GFP at the linker N‐terminus and a BFP at AAA2 to detect linker movement in a dynein AAA1 Walker‐B hydrolysis mutant.^35^ The EM study showed further that the dynein mutant ATP state is very similar to the wildtype dynein ATP‐hydrolysis transition state, trapped using ADP‐vanadate (ADP.Vi).^34^ This suggests that these two states are globally similar and that a higher resolution dynein crystal structure in the ADP.Vi state^36^ (dynein‐ADP.Vi) can be used to analyse the ATP induced conformational changes (Figure 2B).

In dynein‐ADP.Vi, the AAA+ ring is closed and the tripartite interface between AAA1L, AAA1S and AAA2L is fully formed. The AAA1L sensor‐I loop has swung in to contact AAA2L to further stabilize the closure of the AAA1 site. The adenine base is bound by multiple, mainly hydrophobic contacts in the pocket between AAA1L and AAA1S. The ADP phosphates bind the AAA1L Walker‐A and ‐B motifs. The vanadate moiety is stabilized by the AAA2L arginine finger, the AAA1L Walker‐A lysine, the Mg2^+^‐coordinated by the AAA1L Walker‐B motif, the AAA1L sensor‐I asparagine, and the AAA1S sensor‐II arginine.

The AAA1 site of the dynein motor only closes in the presence of ATP. ADP binding is not able to induce this conformational rearrangement,^18^ which indicates that the ATP γ‐phosphate group has a crucial role in the closure of the AAA1 site. The residues contacting the vanadate moiety in the dynein‐ADP.Vi structure are therefore key candidates that could drive the closure of the AAA1 site. The actual trajectory of the AAA2L and AAA1S domains during the movement towards AAA1L remains to be established. The AAA2L and AAA1S positions seen in the various dynein structures^16^, ^17^, ^18^, ^21^, ^26^, ^34^, ^36^ might represent snapshots of this trajectory.

The fact that a closed AAA1 site is also observed in the EM structure of the ATP state^34^ suggests that most of the contacts between the nucleotide and the AAA1 site are formed upon ATP‐binding. However, a high resolution structure of the ATP‐state will be required to assess if and how it differs from the ATP‐hydrolysis state.

**Figure 3:**
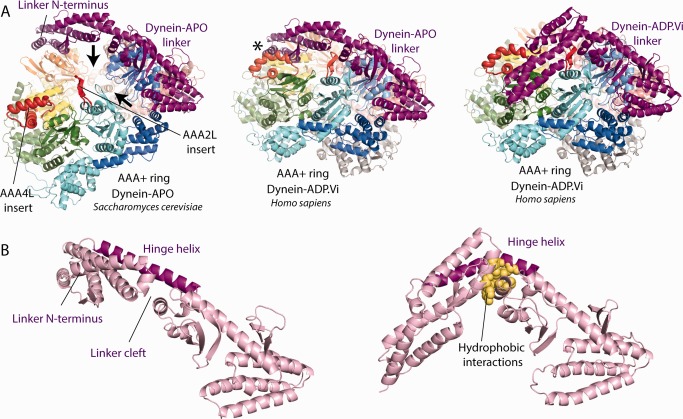
Linker bending upon ATP‐binding to AAA1. **(A)** Model for linker bending based on the currently available crystal structures of the dynein‐APO and dynein‐ADP.Vi states. Left panel: The gaps between AAA1L/AAA2L and AAA5L/AAA6L (black arrows) create an AAA2‐AAAA5 block of AAA+ domains. The AAA4L and AAA2L inserts are highlighted in red. Middle panel: This block moves towards AAA1L when ATP binds to the AAA1 site. The resulting closure of the AAA+ ring leads to a steric clash between the AAA4L insert and the linker N‐terminus (asterisk). Right panel: The clash is resolved by the linker N‐terminus switching into the bend conformation. In addition to the AAA4L insert, an insert in AAA2L is also important for linker bending. **(B)** The different linker conformations. Left panel: The straight linker. The hinge helix spans the central linker cleft. Right panel: In the bent linker conformation the hinge helix is distorted. Hydrophobic interactions across the linker cleft stabilize the bent conformation.

Insights into the question of how ATP‐binding to AAA1 leads to linker bending can be obtained by comparing the dynein‐APO and dynein‐ADP.Vi crystal structures.^36^, ^37^ In dynein‐APO, AAA2 to AAA5 form a block. When the AAA1 site closes around ATP, the whole block moves towards AAA1L. This results in the closure of AAA+ ring which would lead to a steric clash between the AAA4L insert and the N‐terminus of the straight linker (Figure 3A). The clash is relieved by the linker bending at its central cleft.^36^ Consistent with this idea, deletion of the AAA4L insert severely affected linker remodelling.^36^ A recent EM^34^ study appears to have trapped a dynein conformation in which the ring is closed, but the linker is only partially bent. In this state the N‐terminus of the linker is close to AAA4, which is consistent with the steric clash model presented above.

An insert in AAA2L is also important for the ATP induced linker remodeling.^18^ FRET‐studies have demonstrated that the deletion of this insert prevents linker bending.^18^ In the dynein‐ADP.Vi structure, the AAA2L insert contacts AAA1L and stabilizes the closed AAA1 site. The deletion of the AAA2L insert may interfere with the closure of AAA1 and thus only indirectly prevent linker bending. However, it is also possible that the AAA2L insert may play a more direct role as originally hypothesized.^18^, ^21^, ^26^

When the linker bends the hinge helix, which spans the central linker cleft,^36^ is distorted. Hydrophobic interactions across the cleft^36^ counteract the mechanical strain caused by this distorted helix and stabilize the bent linker conformation (Figure 3B). The reported variability in the position of the linker N‐terminus suggests that there is a fine balance between these opposing forces.^32^, ^34^

**Figure 4:**
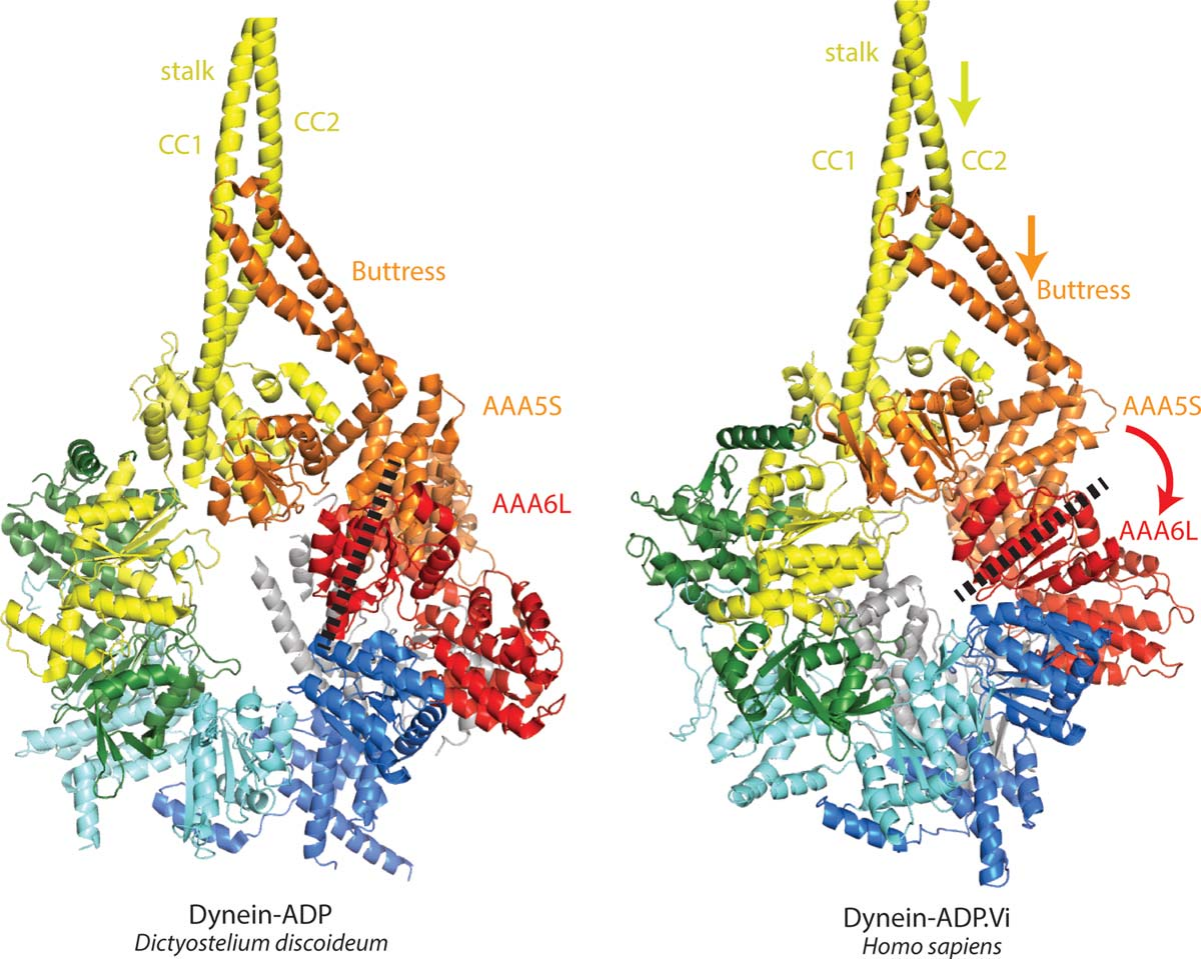
Stalk helix sliding to release the dynein motor from the microtubule. Left panel: Dynein‐ADP. When the stalk is in a conformation that supports the strong microtuble affinity state of the MTBD, the two helices of the stalk (CC1 and CC2) are in a straight conformation at the stalk/buttress interface. Right panel. Dynein‐ADP.Vi. A sliding movement of CC2 with respect to CC1 (yellow arrow) leads to a bulging of CC2 and induces the switch to the low microtubule affinity state of the MTBD. The sliding is induced by a movement of the buttress (orange arrow) that pulls on CC2. This movement of the buttress is caused by a rotation of AAA6L/AAA5S (red arrow), a consequence of the closed AAA+ ring conformation upon ATP binding to AAA1. The dashed line indicates the orientation of AAA6L.

ATP‐binding to AAA1 is also largely responsible for dynein release from microtubules.^38^ Elegant biochemical experiments have demonstrated that the necessary change in MTBD microtubule affinity is driven by the stalk. The two coiled‐coil α‐helices of the stalk can slide with respect to each other and adopt conformations that support the high or low MTBD microtubule affinity state, respectively.^39^, ^40^ The dynein‐APO structure was obtained with a construct where the stalk had been deleted, but a crystal structure of the dynein‐ADP state^18^ (dynein‐ADP) shows a stalk that supports the high microtubule affinity state. Comparing dynein‐ADP with dynein‐ADP.Vi, where the stalk supports the low MTBD microtubule affinity state,^36^ provides insights into the ATP induced stalk helix sliding. Although the stalk and stalk‐interacting regions in the dynein‐ADP.Vi structure are at low resolution, overall conformational changes between the two structures can still be analysed. The closure of the AAA+ ring upon ATP‐binding to AAA1 forces AAA6L and AAA5S, which are tightly associated, to rotate. This rotation in turn drives a movement of the buttress, extending from AAA5S, relative to the stalk, which induces the sliding movement of the stalk coiled‐coil helices.^36^ The subsequent conformational change in the MTBD^41^ causes the release of the dynein motor from the microtubule (Figure 4). An interesting open question is whether microtubule release occurs before the remodelling of the linker or if these two events are simultaneous.

## THE MECHANISM OF ATP‐HYDROLYSIS AT AAA1

**Figure 5:**
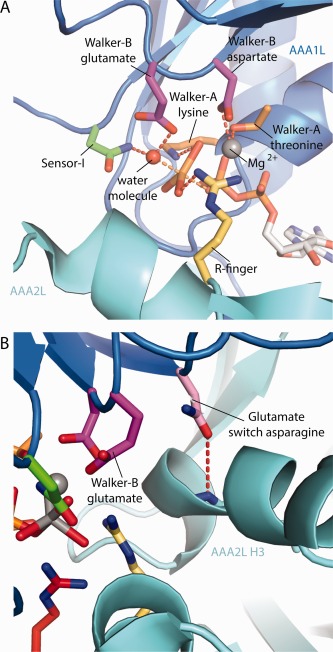
Mechanism of ATP‐hydrolysis at the AAA1 site of the dynein motor. A: Mechanistic model based on the dynein‐ADP.Vi structure. A water molecule is positioned by the sensor‐I asparagine and deprotonated by the Walker‐B glutamate for the subsequent nucleophilic attack onto the γ‐phosphate of the ATP. The transition state is stabilized by contacts with the arginine finger, the Walker‐A lysine and the catalytic ${\text{Mg}}_{.}^{2+}$ B: Potential glutamate switch in the AAA1 site. The ability of the Walker‐B glutamate to deprotonate a water molecule might be influenced by a glutamate switch asparagine. In the dynein‐ADP.Vi structure this residue interacts with AAA2L H3, allowing the Walker‐B glutamate to adopt its hydrolysis competent conformation.

The dynein‐ADP.Vi crystal structure^36^ shows the AAA1 site trapped in the process of ATP‐hydrolysis. The position of the sensor‐I asparagine, which positions a water molecule for the nucleophilic attack onto the γ‐phosphate, and the Walker‐B glutamate, which activates this water molecule by deprotonation, strongly suggests that the nucleophilic attack proceeds via an associative SN2‐like mechanism (Figure 5A). This mechanistic model is in accordance with earlier kinetic work on the dynein motor domain that determined the chirality of isotope labelled γ‐phosphate after hydrolysis.^42^ The arginine finger, the Walker‐A lysine as well as the catalytic Mg^2+^, which is coordinated by the Walker‐B aspartate and the Walker‐A threonine, stabilize the pentacovalent transition state of the γ‐phosphate.

The AAA1 active site of many dyneins harbours an asparagine residue that may hydrogen bond to the Walker‐B glutamate to form a so called glutamate switch. In other AAA+ proteins,^43^ an equivalent asparagine binds to the Walker‐B glutamate upon ATP‐binding. In these cases hydrolysis is prevented because the glutamate is unable to activate a water molecule for nucleophilic attack. Subsequent binding of protein partners to the AAA+ machine disrupts the glutamate switch and activates hydrolysis.

The glutamate switch has not yet been observed in dynein as there is currently no high resolution structure of the ATP state available. We speculate that the initial stages of ATP‐binding lead to the formation of the glutamate switch. In this way, the dynein motor could gain time to release from the microtubule before the ATP‐hydrolysis cycle is completed to prevent an unproductive power stroke. Although it is not clear how the subsequent disruption of the glutamate switch is triggered, there are several possible scenarios. The conformational changes in the MTBD that cause the release from the microtubule might indirectly trigger such an event. Alternatively, the disruption of the glutamate switch could be controlled within AAA1 site itself. While ATP‐binding and the initial stages of AAA1 site closure might lead to the formation of the glutamate switch, the complete AAA1 closure might cause its disruption. It is also possible that there is no need for an external signal controlling glutamate switch disruption. The switch itself might have a limited lifespan, only delaying the dynein motor cycle to ensure that microtubule release occurs before ATP‐hydrolysis.

How the conformation of the glutamate switch looks like after disruption is revealed by the dynein‐ADP.Vi structure,^36^ which shows the AAA1 active site in the transition state of ATP‐hydrolysis. In this structure, the potential glutamate switch asparagine is angled away from the Walker‐B glutamate by an interaction with a main‐chain carbonyl oxygen at a prominent bend in the AAA2L H3 helix (Figure 5B). The glutamate switch asparagine is almost universally conserved in all dynein isoforms except cytoplasmic dynein‐1, where it is substituted by a cysteine in about 40% of cases (including *Dictyostelium discoideum*). It will be interesting to ask if this cysteine can participate in the glutamate switch interaction.

## PHOSPHATE RELEASE AND THE DYNEIN POWER STROKE

**Figure 6:**
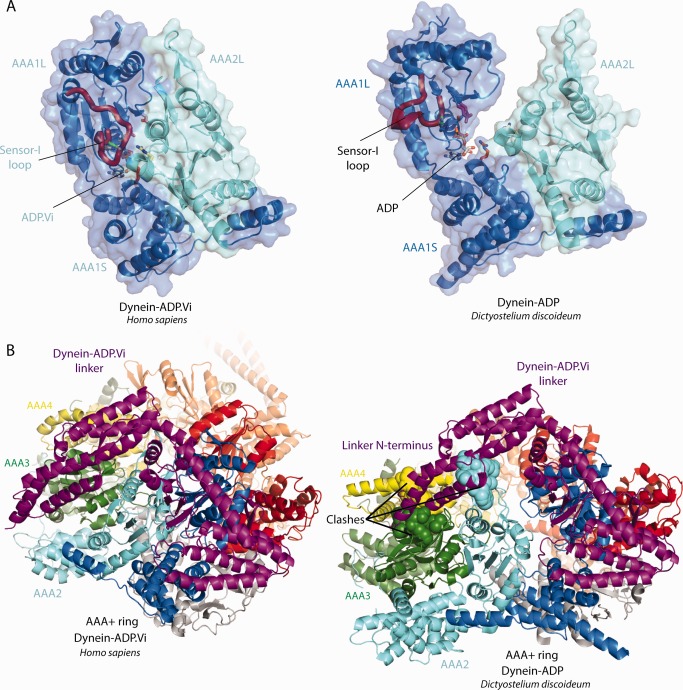
Phosphate release and the linker powerstroke. A: Phosphate release after ATP‐hydrolysis. Left panel: In dynein‐ADP.Vi, the AAA1 nucleotide binding site is completely shielded from bulk solvent. Right panel: In dynein‐ADP, the AAA1 site has opened up and the sensor‐I loop has moved away from the nucleotide binding site. Both of these conformational changes on its own would offer a potential escape route for the phosphate. B: Steric clash model for the linker powerstroke. Left panel: In the dynein‐ADP.Vi structure, the linker is bent and the AAA+ ring is closed. Right panel: Superimposing the bent linker of dynein‐ADP.Vi onto the dynein‐ADP AAA+ ring leads to steric clashes (spheres) between the linker N‐terminus and the AAA2‐AAA4 part of the ring. These clashes might perturb the hydrophobic interactions across the linker cleft, which stabilize the bent linker conformation, to trigger the linker powerstroke.

Kinetic and FRET studies suggest that phosphate release occurs before microtubule rebinding and the power stoke of the dynein motor.^28^, ^35^ Insights into the order in which these events occur can be obtained by comparing the dynein‐ADP.Vi^36^ and dynein‐ADP^18^ structures. In dynein‐ADP.Vi, there is no obvious escape route for the phosphate because the vanadate is completely shielded from solvent. In dynein‐ADP, the sensor‐I loop has moved away from AAA2L and the AAA1 site has opened up (Figure 6A). Both these conformational changes would create escape routes for the phosphate. Do they occur simultaneously or does the sensor‐I loop rearrangement happen first, similar to the well‐established back‐door mechanism for phosphate release in the myosin motor^44^? We favour the latter the scenario, because compared to an opening of the AAA1 site, the sensor‐I loop rearrangement would only marginally disturb the overall AAA+ ring geometry. This would preserve the bent linker conformation and therefore be in accordance with the aforementioned kinetic and FRET studies.

After phosphate release, the dynein motor rebinds to the microtubule and undergoes a powerstroke. How might this straightening of the linker be triggered? Although there is currently no definitive answer to this question there are several possible scenarios. The comparison between the dynein‐ADP.Vi and dynein‐ADP structure suggests that microtubule rebinding causes the AAA1 and AAA4 sites to open up, which splits the AAA+ ring into an AAA2‐AAA4 and an AAA5‐AAA6 + AAA1 block. Superimposing the bent linker observed in dynein‐ADP.Vi onto the split dynein‐ADP ring suggests, that the linker N‐terminus would clash with the AAA2‐AAA4 block (Figure 6B). This could perturb the hydrophobic interactions across the linker cleft leading to it returning to its default straight conformation.^32^ The straightening of the linker is likely driven by releasing the strain in the hinge helix.^36^ Another suggestion for triggering the powerstroke is based on the idea that the bent linker conformation has to be actively maintained by contacts with the AAA+ ring. The removal of these contacts upon AAA+ ring opening would allow the linker to adopt the straight conformation.^31^

## THE RELEASE OF ADP

**Figure 7:**
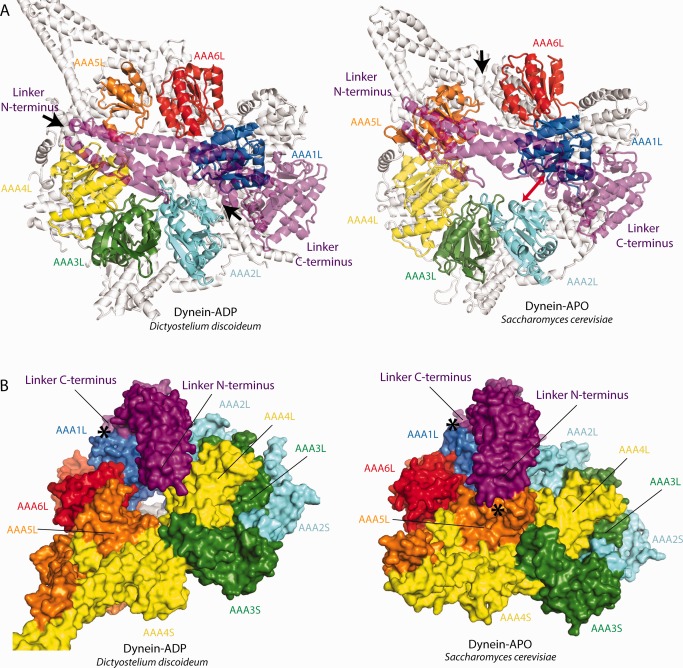
The release of ADP from the AAA1 nucleotide binding site. A: Left panel: In the AAA+ ring of dynein‐ADP there are gaps between AAA1L/AAA2L and AAA4L/AAA5L (black arrows). Right panel: In dynein‐APO, AAA5L has moved toward AAA4L creating a gap between AAA5L/AAA6L. The gap between AAA1L/AAA2L has further widened (red double arrow) to allow the release of ADP. For clarity all AAASs are shown in white and the linker is represented as transparent cartoon. B: Conformational changes induced by the shift of AAA5L. Left panel: Dynein‐ADP structure. Right panel: Dynein‐APO structure. The movement of AAA5L from AAA6L towards AAA4L allows the linker‐N terminus to dock onto AAA5L. The linker C‐terminus is tightly associated with AAA1L. In contacting AAA1L and AAA5L at the same time, the linker acts as a spacer that pushes the AAA1 + AAA6 and AAA2‐AAA5 blocks of the AAA+ ring apart to further open up the AAA1 nucleotide binding site. The docking sites of the linker N‐ and C‐terminus have been marked with black asterisks.

After the powerstroke, ADP has to be ejected from the AAA1 site. Comparing the dynein‐ADP and dynein‐APO structures suggests that this is achieved by further widening the AAA1 site. AAA5L seems to play a key role in this conformational change. In the dynein‐ADP structure, AAA5L is close to AAA6L and the linker N‐terminus contacts the AAA2‐insert.^18^ In the dynein‐APO structure, AAA5L contacts AAA4 to create two blocks of AAA+ domains (AAA5‐AAA2 and AAA6 + AAA1) (Figure 7A). The new position of AAA5L is stabilized by the linker N‐terminus which firmly docks onto it^37^ (Figure 7B). The linker C‐terminus is tightly associated with AAA1L (Figure 7B), which allows the linker to act as a spacer that pushes the two blocks apart to widen the AAA1 site^21^.

## CONCLUSIONS

The recent dynein crystal and EM structures have allowed us to gain unprecedented insights into the mechanism of one of the most complex AAA+ machines nature has evolved. Unlike other AAA+ proteins, the dynein AAA+ ring has two substrates that it remodels: the linker and the stalk coiled‐coil. The co‐ordinated remodelling of these two substrates during the ATP‐hydrolysis cycle allows dynein motors to generate efficient movement. Although we have made progress in understanding of how ATP‐hydrolysis at AAA1 causes these remodelling events, there are still a number of important open questions. What does the ATP state of the dynein motor look like? What are the molecular interactions at the stalk/buttress interface that drive the sliding of the stalk helices and lead to the change in microtubule affinity? Does linker bending occur in one step or are there discrete substeps? How does microtubule binding trigger the powerstroke? Crystal and electron microscopy structures at higher resolution are needed to answer these questions.

The structural analysis presented in this review is based on crystal structures from different dynein isoforms from *Saccharomyces cerevisiae*, *Dictyostelium discoideum,* and *Homo sapiens*. Also some of the dynein motor states represented by these crystal structures, like dynein‐APO or dynein‐ADP, would naturally occur when bound to the microtubule. We can therefore not exclude the possibility that some of the conformational changes observed in the structures are isoform or species specific or that additional conformational changes might occur upon microtubule binding. However, the basic structural elements needed to enable the dynein motor to walk along microtubules are conserved in all dyneins, making it likely that the observed structural changes apply for all dyneins.

Taken together, huge progress has been made in our understanding of the basic dynein motor mechanism. The field is currently approaching the “next frontier” which is how this basic mechanism is influenced by important dynein motor regulators like Nudel/Lis1 or the dynactin complex so that dynein motor activity can be exactly shaped according to the biological function.^31^, ^45^
